# Decision-Making Model for Addressing Role Conflict for Psychology Trainees When Supporting Family and Community

**DOI:** 10.3389/fpsyg.2021.745368

**Published:** 2021-11-01

**Authors:** Natalie A. Larez, Jill D. Sharkey

**Affiliations:** Department of Counseling, Clinical, and School Psychology, University of California, Santa Barbara, Santa Barbara, CA, United States

**Keywords:** psychology graduate students, training programs, ethics, role conflict, equity

## Abstract

As the field of psychology continues to make efforts to diversify the field, training programs must adapt to include the needs of diverse students. Universities in the United States mirror middle-class norms and values, which implies that students are expected to separate from familial roles and focus on their personal growth. This conflicts with core values and intentions of students from collectivist cultures. Although psychology trainees are obligated to adhere to professional ethical standards, a growing number of psychology trainees from collectivistic cultures need support to manage role conflict within potentially ambiguous standards regarding how to care for family and community members. This need is further complicated when training programs consider the lack of equitable access to mental health care resources in communities where their psychology trainees come from. In this paper, we engage in ethical decision making to address two scenarios representing role conflict between training program expectations and collectivist community and familial obligations. Through this exercise we develop and propose a Decision-Making Model for Addressing Role Conflict for Psychology Trainees. This conceptual model details a novel framework to assist psychology trainees when addressing the mental health of family and community while also providing guidance to help graduate training programs proactively equip their students with the skills and ethical framework they need to balance role conflicts such as when family and community members desire and need mental health support.

## Example Scenario: Family

Alejandra is a graduate student completing a doctoral degree in school psychology in a different state from her hometown. Alejandra is a first-generation college graduate and is the first in her family to pursue a doctoral degree. She grew up in a low-resourced, rural, and border town community. Her community has limited access to health care, mental health services, and educational opportunities.

During her 1st year of graduate school, Alejandra’s younger cousin disclosed that he was thinking about suicide. His pseudonym will be Andres. Andres shared this with Alejandra on their drive back to his house in their hometown. Alejandra and Andres parked at a local park to talk about Andres’ recent disclosure of thoughts of suicide. Alejandra asked Andres if he had a plan or the means to follow through with his plan. She also asked Andres if he had mentioned it to anyone else in their family or a professional at school. Andres responded with, “No, I don’t know who to tell.”

After the conversation with Andres, Alejandra and Andres came to a decision to tell his mom together upon their arrival. Alejandra drove him home and spoke to Andres and his mother in the same room and discussed the lethal means available to him. Andres and his mother discussed the removal of the means of suicide that Andres was thinking about utilizing. Following this conversation, Andres’ mother took him to a larger city, 2 h away, to receive mental health services every 2 weeks.

In the years to follow, Andres still speaks to Alejandra about his mental health concerns. On several occasions, when Alejandra returns to their hometown and they are driving, Andres breaks down in the car about his high anxiety and current life circumstances related to other health concerns. Alejandra speaks to Andres and provides coping skills support and assures that Andres is feeling supported by his current therapist. To date, Andres has made immense progress.

## Example Scenario: Community

During Alejandra’s 2nd year of her doctoral program, a close community member’s nephew died of suicide. His pseudonym will be Carlos. Carlos was a 19-year-old male who had lost his father to a tragic death when he was a young child. Carlos was also experiencing frequent disputes with his close family members. As shared by his girlfriend and his suicide note, he had a happy relationship with his girlfriend. Carlos had never received mental health services.

Alejandra was in her home state on the night of his death. During this time, Alejandra felt an obligation to drive to her hometown to console the family and community members who were immediately affected. Upon deciding, Alejandra’s brother, who is a medical student, reminded Alejandra that she had limited competence and skills. Alejandra did not drive to support the family and instead sent the family several messages via social media. Alejandra consoled her friend, the close community member, in person. The family did not receive any mental health services following the death of Carlos.

In her roles as a psychology trainee, older sister, and community member of a rural low-resourced community, Alejandra demonstrates how professional ethical standards, community and family obligations, and health disparities interact to create an ethically challenging scenario for first-generation and partially trained psychologists.

## Introduction

Psychology training programs offer guidance, supervision, and practice in regard to providing services for patients in their care. However, minimal research has been conducted examining the frequency, method, repercussions, and potentially positive effects of psychology trainees utilizing their skills prior to completion of their doctoral program and gaining the credentials and/or licensure they need to practice. For example, a psychology trainee may be contacted by a family member who discloses suicidal thoughts, creating a dilemma for the psychology trainee and leaving them to navigate whether they should proceed to safety plan with the family member or refer them to a community organization that may or may not be able to provide immediate support. A family or community member may also seek support from the psychology trainee, which may lead to a psychotherapy-like session. In a separate scenario, a psychology trainee may be contacted in the event of a community crisis and struggle with how to balance their competence and dual roles in providing much-needed support. In the aforementioned scenarios, the psychology trainee must manage seemingly conflicting roles, information, and ethical obligations.

With this novel application of role theory, in the context of psychology trainees and community and family care, we provide an overview of a theoretical framework that can help support a psychology trainee when they experience role conflict. Although licensed psychologists likely also experience role conflict, we focus here on psychology trainees and training programs as a way to address role conflict from the foundations and development of practice. We provide empirical literature to explain how role theory has been used in the past and propose utilizing an intersectional lens to acknowledge the complexity of identity among a growing number of diverse psychology trainees. Additionally, we outline potential equity considerations intended to encourage conversations to address role conflict among training programs and psychology trainees. The list is not intended to be exhaustive, rather some starting points for discussion. We follow by providing an adaptation of an ethical decision-making model as a tool for training programs to utilize in supporting their students who may be experiencing role conflict and ambiguity. We end the paper with recommendations for training programs and future directions for further research in this area.

### Theoretical Framework

#### Role Theory

Work-family theories have been grounded in Bronfenbrenner’s system’s theory, Ecological Theory. Bronfenbrenner (1977) proposed that individual components of systems and individuals are likely to affect and interrelate to each other, highlighting that no system is independent of each other. Role theory is often used to understand the various roles a person plays in their environments and the groups they belong to Kahn et al. (1964). Each member of a person’s various environments, such as supervisors, colleagues, and family members, has role expectations that influence their behavior. A role conflict occurs when various members of the focal person’s various environments hold different expectations and in turn, create potentially conflicting circumstances such that compliance with one would make compliance with the other more difficult (Kahn et al., 1964). Competing role expectations may lead to role ambiguity. Role ambiguity occurs when information is communicated inadequately or not at all and can lead to a person not performing to the role expectations (Kahn et al., 1964).

Role theory has yet to be used as a tool to understand role conflict and role ambiguity for psychology trainees in the context of community and family support. Our application of role theory to psychology trainees is novel and untested, but can be supported by previous empirical literature that has found role conflict and role ambiguity to have effects on employer well-being. Acker (2003) examined how role ambiguity and conflict can predict the burnout of mental health service providers who function under the bureaucracy of mental health care organizations. Researchers found that both role conflict and role ambiguity were positively correlated with factors of burnout such as emotional exhaustion and depersonalization (Acker, 2003). Dasgupta (2012) also found that an increase in role overload, role conflict, and role ambiguity led to higher rates of disengagement and exhaustion among nurses in India. Thus, addressing the potential for role conflict, particularly when it causes a conflict within professional ethical standards, is important to address in order to help develop a diverse, engaged, and thriving workforce of licensed psychologists.

Considering role conflict in the context of the example scenarios, psychology trainees may experience opposing expectations from their training program’s ethical standards and familial and/or community obligations. The lack of guidance from credentialing entities, such as the American Psychological Association (APA) and the National Association of School Psychologists (2010; NASP), may contribute to difficulty reconciling various expectations. In the context of supporting community or family members in an informal setting, role ambiguity may arise due to the lack of guidance from the credentialing entities and program guidelines; psychology trainees may not have sufficient information to perform in their role/s appropriately.

#### Intersectionality

Intersectionality has often been used to conceptualize how race, gender, socioeconomic class, and other social identities create different systems of oppression. We utilize intersectionality in order to acknowledge the complexity of diversity in psychology trainees and the various intricacies that may affect the way in which psychology trainees experience a field that is based on Euro-American norms. The term *intersectionality* was originally coined by Kimberleì Crenshaw in the context of antidiscrimination laws not protecting Black women given that the laws treated gender and race as mutually exclusive categories for determination (Crenshaw, 1989). The original work of intersectionality challenged the use of the “single-axis” framework, which only considers one, rather than multiple, forms of identity. The intersectionality conceptual framework has since been used in multiple fields of study to continue to understand various social identities and their interaction with the environment. With the support of role theory, the intersectionality conceptual framework is useful in understanding the multiple identities of psychology trainees and their various roles and expectations within the field and their communities.

The intersectional conceptual framework has previously been used in the field of psychology in multiple areas, such as, the psychology of women (Warner et al., 2018), the discrimination of school psychology graduate students (Proctor et al., 2017), and generally challenging the field (Overstreet et al., 2020; Grzanka et al., 2020). Additionally, it has been utilized as a framework for treatment (Clauss-Ehlers et al., 2019). Previous research has indicated that role ambiguity, work-family conflict, and role conflict are all moderated by the gender of the individual (Boles et al., 2003). This suggests that role conflict among psychology trainees may also be experienced differently depending on the trainee’s gender. The intersectional conceptual framework has yet to be used as a framework to understand the various obligations, roles, and identities of psychology trainees when considering the various environments of their training program and the communities they belong to. We argue that an intersectional framework and role theory can offer important insight to this unique nexus of identities and environments of psychology trainees.

### Equity Considerations

Universities in the United States mirror middle class norms (Stephens et al., 2012), creating a potential role conflict for students who are navigating a training program in the United States with cultural values that may not align with university expectations. Further, the field of professional psychology is based on Euro-individualistic perspectives of well-being, coping, care, and recovery and ethical standards are designed to respect “the rights of individuals to privacy, confidentiality, and self-determination” (American Psychological Association [APA], 2017a). Meanwhile, 45% of psychology trainees are from underrepresented racial and/or ethnic groups (American Psychological Association [APA], 2020). Considering the growing diversity of the field of psychology and the various cultural views, community care models, and lived experiences that psychology trainees are entering the field with, it is imperative that the field of professional psychology challenge the Euro-American and individualistic models of well-being and training to consider perspectives of other cultural values, especially those who value collectivist ideals.

#### Collectivism, Individualism, and Culture

Interdependence and intention to maintain familial roles while in higher education is important for students from collectivistic cultures. Collectivism and Individualism are two psychological constructs that are attributed to different socialization patterns and values across cultures (Oyserman et al., 2002; Triandis, 1989). Notably, these socialization practices heavily influence a person’s behavior toward their community and family (Triandis, 1989). Individualism is conceptualized as a worldview that centers personal goals, person distinctiveness, and personal control (Oyserman et al., 2002). Collectivism underscores the importance of group membership and the core traits of sacrifice for the common good and maintaining harmonious relationships (Oyserman et al., 2002). Psychology trainees who attend graduate training programs with a worldview of collectivism may feel obligated to utilize their skills for their community to maintain familial/community roles, especially when there is significant need due to lack of resources.

#### First Generation College Students

First generation college students are often in familial roles that lead to greater frequency of practicing skills they learned from college with their families and communities. Researchers found that in comparison to their continuing-generation counterparts, first-generation college students had more interdependent motives for attending college (Stephens et al., 2012). First-generation and low-income students often attend university settings with the focus of *maintaining* familial roles (Covarrubias et al., 2019). Although much of the research focused on first-generation familial obligations has been conducted with undergraduate students, the premise of maintaining familial and community obligations may still persist as first-generation students progress through and attain graduate degrees, such as doctoral degrees in applied psychology. Interdependence may lead to role conflict for first-generation and/or low-income students.

#### Rural Communities

In addition to the potential higher frequency of this ethical challenge among psychology trainees who are first generation college students and/or from collectivistic cultures, APA highlighted the growing need for mental health support in under-resourced and rural communities (2017b). For example, people in poverty experience depression at 2.5 the rates of their counterparts (American Psychological Association [APA], 2017b). Additionally, although ethnic minoritized populations are just as or more likely to experience mental health disorders, they receive considerably less treatment. American Psychological Association [APA] (2017b) shared that 18.7 percent of rural residents have a diagnosable mental health disorder but have low access to mental health support exacerbated by the chronic shortage of mental health professionals in rural America. With this in mind, rural communities may have limited resources available to support the mental health of psychology trainee’s family and community members. When these pieces of intersectionality arise, the challenge of navigating limited guidance from psychology training programs, first generation college student status, and inequitable mental health care access are compiled for a growing number of diverse psychology graduate students.

#### Community Health Workers

Given the limited quantity and accessibility of quality mental health professionals in rural and/or lower-resourced communities, psychology trainees from these communities may serve a unique role in between the role of a lay Community Health Care Worker (CHW) and a licensed mental health professional. The American Public Health Association (APHA) has officially defined CHWs as “CHW as a “frontline public health worker who is a trusted member of and/or has an unusually close understanding of the community served” (American Public Health Association [APHA], 2016). There is reason to believe that psychology trainees who are from these communities may also serve as a trusted member of the community and therefore, be able to leverage their unique knowledge about the community in an effort to better support community members. When CHWs are used in parallel with Primary Care Providers (PCP), favorable intervention outcomes have been found (Waitzkin et al., 2011). There is limited research on how and to what effect a psychology trainee may fill a similar role as CHWs given they have evolving training that is increasingly more advanced than a CHW.

Rhodes and Langtiw (2018) highlight the need of the field of psychology to shift to community-based approaches to mental health rather than the dominant individualistic approach. In community-based approaches, there is a focus on models of care beyond the individual person and extends to familial and community processes. Moreover, we argue that training programs should also consider how psychology trainees engage in their own community care and possibly maintain familial roles of healing as someone who has training beyond a lay person. Because folks from collectivistic, first-generation, and other cultural identities may enter a training program with intentions of maintain familial roles or with high levels of group membership, an individualistic and Euro-American approach of psychology training programs may cause role conflict and ambiguity if expected to maintain strict boundaries between their dual roles.

These equity considerations were integrated to conceptualize the various factors that may be contributing to the perceived role conflict of psychology trainees. Although we cannot capture the full complexity of culture, middle-class university norms, and Euro-American professional psychology care models, we argue that these equity considerations support fruitful dialogue among training programs and the field of psychology. We believe that world views (i.e., individualism vs. collectivism), cultural perspectives, socioeconomic status, available community resources, first-generation status, gender, and immigration status are some factors that can create perceived role conflict and ambiguity among psychology trainees. To address this novel application of role conflict to psychology trainees, we developed a decision-making model to support psychology trainees as they are faced with competing role expectations.

### Selected Decision-Making Model

In order to provide guidance related to this ethical challenge, a seven-step model was adapted. The seven-step model was proposed by Jacob et al. (2010) and outlined the following steps: (1) Identify the problem; (2) identify the potential legal and ethical concerns; (3) consult ethical guidelines; (4) consult with other professionals and experts in the field/s; (5) evaluate the rights and responsibilities of all parties; (6) consider alternative solutions; and (7) make a decision and assume responsibility. This model has been regularly applied when addressing ethical dilemmas in applied psychology (e.g., Sharkey et al., 2017; Stein and Sharkey, 2015). Additionally, it is important that psychology training programs acknowledge that the decision-making process is not universally applicable. LeFebvre and Franke (2013) found that variation in cultural traits, societal traits, and cultural setting of a situation had an impact on the decision-making process. Yates and de Oliveira (2016) further describe how important phases of decision-making are addressed in different cultures. Therefore, it is clear that decision-making models intended for psychologists to utilize in practice, should also be adapted to align with the growing diversity needs of the field.

We adapted this ethical decision-making model to consider the role of the training program in steps 4–7 as critical for providing proactive training, responsive supervision, and conducting ongoing research and evaluation to help their trainees resolve these ethical dilemmas. Through this process we developed a Decision-Making Model of Addressing Role Conflict for Psychologists in Training.

## Describing the Ethical Challenge

Psychologists receive a variety of training in skills such as, collaboration, assessment, problem solving, mental health services, crisis management, and helping skills. In low-resourced and/or rural communities, there is less accessibility to trained personnel who have a skillset in these areas. Thus, psychology trainees may be asked to utilize their skills prior to completing their credential program and/or outside their professional setting. This creates a dilemma for the psychology trainee to either support family and community members without being licensed or to leave family and community members with little to no support during a crisis. Given a lack of guidance in training programs and ethical standards when navigating dual roles in under-resourced and marginalized communities, this paper will focus on the ethical considerations necessary in order to navigate scenarios in which psychology trainees feel the obligation to support their community. A better understanding of this process will inform a decision-making model for addressing role conflict to support psychology trainees who come across similar scenarios.

## Legal and Ethical Guidelines

Psychology trainees who find themselves in a scenario in which their skills may be helpful should consider the safety, ethical, and legal implications for proceeding in supporting their community and family members outside of their professional work setting. To guide the ethical decision-making process, psychology trainees should review the American Psychological Association’s (APA) Ethical Principles, the National Association of School Psychologists (2010; NASP) Principles of Professional Ethics, and relevant corresponding state laws. The ethical principles and standards most relevant to this dilemma in regard to APA, include Principle A. *Benefice and Non-maleficence*, Principle B. *Fidelity and Responsibility*, and Standard 2. *Competence*. The NASP principles and standards that are most relevant in this ethical challenge are Principle II, *Competence and Responsibility*, Principle III, *Honesty and Integrity in Professional Relationships*, and Principle IV, *Responsibility to Schools, Families, Communities, the Profession, and Society.* Given that many of the standards and principles parallel each other, we have created four categories to concisely describe the obligations of psychology trainees in the context of this ethical challenge.

### Competence

Generally, competence is an important consideration in any scenario when working with patients as a psychologist. In scenarios where there is no formal consent or intake process, it might be more difficult to share boundaries of competence as a psychologist and limitations to confidentiality. In some scenarios, family and community members may be under extreme distress and there may be safety concerns for themselves, and competence may not be the first priority. APA Standard 2, *Competence*, specifically outlines providing services in case of emergencies, “In emergencies, when psychologists provide services to individuals for whom other mental health services are not available and for which psychologists have not obtained the necessary training, psychologists may provide such services in order to ensure that services are not denied (p. 5).” Similarly, NASP Principle II, *Competence and Responsibility*, underscores the importance of school psychologists acting to benefit others within their competence. Psychology trainees are responsible for providing services that ultimately benefit the surrounding community, as long as they are within a psychology trainee’s competence. By implementing these ethical standards, psychology trainees should be able to support family or community members with boundaries and after assessing the immediacy and benefit versus harm that may arise in a scenario. Psychology trainees should also consider if on-going care is necessary and seek further resources for referral.

Ethical standards suggest that psychologists must end services when an emergency is over. Mental health professionals should consider whether the situation warrants emergency use of their skills and proceed with caution, especially when supporting their own family members. APA standard 2 notes that psychologists may proceed with services in an effort to *ensure* that people involved are getting support, which ultimately allows for flexibility for this ethical challenge. Nonetheless, this standard does not address the complexities that arise when communities are low-resourced and may not have other forms of accessibility; it remains unclear how a psychology trainee should proceed when ongoing care is needed, and no other resources are available.

### Fidelity and Integrity

APA Principle B, *Fidelity and Responsibility*, highlights that psychologists also have an ethical responsibility to the community they work with. Acting with fidelity and responsibility means that psychologists should clearly state their obligations and responsibilities in and out of their professional work (American Psychological Association [APA], 2017a). Similarly, NASP Principle III emphasizes the importance of maintaining community trust by clearly stating qualifications and competences. Importantly, psychology trainees may feel the ethical responsibility to support people who are in distress and do not have access to mental health services elsewhere, even if they are not formal clients. However, APA Principle B makes it clear that psychologists must state their obligation to any person they support in a professional capacity and make it clear that they are only providing support given the constraints of the scenario and potential equity issues, such as lack of mental health support in their area. Setting clear boundaries and circumstantial context is important to safeguard the community/family member and the psychology trainee. The psychology trainee and the community/family member should both come to a shared agreement after considering the circumstances if time permits.

In regard to materials and multiple relationships, NASP Principle III highlights that multiple relationships and conflicts of interest may arise. Standard III.4.9 states that school psychologists may not use test materials, or equipment that belongs to the public-school district unless otherwise approved. Thus, school psychologists may not use any materials for community or family members outside their professional setting unless the training school psychologist is able to secure permission beforehand. Importantly, neither NASP standard II nor III provides guidance regarding suicide prevention and intervention, psychological first-aid, or other non-tangible services.

### Beneficence and Responsibility to Communities

APA Principle A, *Beneficence and Non-maleficence*, states that psychologists should aim to provide care that ultimately benefits and minimizes harm to the people and communities they work with. Principle A also underscores the importance of recognizing and safeguarding against the misuse of professional skills and influence on clients and other affected persons (2017). NASP Principle IV, *Responsibility to Schools, Families, Communities, the Profession, and Society*, further adds that school psychologists have a responsibility to promote healthy family, school, and community environments. Principle IV highlights that school psychologists must act proactively to support their environments and maintain a systems-level perspective when working with families. Importantly, standard IV.2.4. states that, “School psychologists may act as individual citizens to bring about change in a lawful manner (p. 12).” In the case of this ethical dilemma, principle IV.2.4. suggests that school psychologists should act with the best intentions for their community, as an individual citizen as long as it is within the confines of the law. By providing support to community and family members, especially in under-resourced communities, school psychologists are providing essential support for their community, school, and families in an effort to challenge the inequities that communities may be facing. When considering both APA Principle A and NASP Principle IV, it is important to consider if providing support to people who are not formal clients benefits them more than it would harm them to deny support.

### Equity and Social Justice

The ethical challenge presented in this paper highlights social justice issues that are systemically entrenched in the mental health services in the United States. The challenge is further exacerbated by the inequitable access to mental health services among underserved and rural communities. Further, this ethical dilemma challenges norms in universities that have often ignored the complexity of being first-generation students and/or students from an under-resourced, minoritized, collectivistic, and low-income community. For these reasons, it is also important to consider APA Principle D, *Justice*, and NASP Standard 1.3, *Fairness and Justice.* APA Principle D states, “Psychologists recognize that fairness and justice entitle all persons to access to and benefit from the contributions of psychology and to equal quality in the processes, procedures, and services being conducted by psychologists (p. 4).” NASP further adds that school psychologists have the responsibility to promote fairness and justice. With these two guidelines in mind and understanding the obstacles currently in place for some communities to access quality services, psychologists may justify their actions in supporting those outside their professional clientele.

### Rights and Dignity

APA Principle E, *Respect for People’s Rights and Dignity* and NASP Principle I: *Respecting the Dignitiy and Rights of All Persons* focus on the rights of individuals for self-determination, privacy, and confidentiality. These principles also include the importance of avoiding multiple relationships if this impairs the psychologist’s objectivity, competence, and/or effectiveness. These principles focus on the individual and their right to autonomy. As such, they may overlook key cultural considerations of collectivism. For example, informed consent is the collaborative process between a psychotherapist and client with a purpose of sharing information about treatment, so the client is able to make a decision about beginning treatment (Lasser and Gottlieb, 2017). The consent process is intended to respect the client’s independence and nurture a shared decision-making process. Clients enter a therapeutic relationship with varied values and cultural norms that may influence how they perceive the consent process. Even so, ethical codes that guide the consent process reflect Western values such as autonomy and individualism (Meer and VandeCreek, 2002). To practice with cultural humility, Meer and VandeCreek (2002) recommend that psychologists discuss the meaning of confidentiality with their clients and in what ways a client wants family members (e.g., elders) to know about and/or be involved in their treatment. In the context of the ethical scenarios presented in this paper, the consent process is further complicated due to the informality of a potential therapeutic relationship. Attending to both ethical considerations and cultural considerations, it seems important for the psychology trainee to address limits to their level of competency, regularly check for consent in proceeding with support, and discuss confidentiality and who in the family or community must (e.g., reporting abuse) or may (e.g., a parent or other guardian who may offer additional support) be told information about the concerns and related support.

## Consultation With Others

Given the immediacy that some scenarios present, consultation with peers, colleagues, or trainers may not be viable prior to acting. For example, immediate action is required if the psychology trainee is in their hometown and their family member discloses suicidal thoughts. In addition, without proactive training and supervision in how to address role conflicts as they arise, consultation may yield conflicting advice and uncertain guidance.

As training programs increase the number of psychology trainees from historically underrepresented groups, such as first-generation college students or who identify with collectivist cultures, it is important to proactively teach ethical standards with the reality of the potential need to support family and community outside training norms. Toward that end, based on equity considerations promoting better attention to family and community roles, training programs may want to integrate course curriculum to include attention to the roles and boundaries of CHW workers and discuss the potential for and decision-making processes to address role conflict. Moreover, discussions around inequitable mental health care access for low-income, immigrant, and/or communities of color should be a topic often discussed in schools and university settings. Providing early training in psychological first aid may help psychology trainees provide competent care to family and community members when needs arise.

Training programs also need to work on their own attention to multicultural strengths and Euro-centric systems and practices may need to be disrupted in order to provide more effective supervision and training. In their development of the Multicultural Integrated Supervision Model, Mitchell and Butler (2021) provide key considerations for supervisors. Supervisors must acknowledge and attend to privilege and power within the supervisory relationship. This includes overtly discussing intersecting identities and how they impact the supervisory relationship. In addition, supervisors must create the space for discussions about culturally responsive practices in order for them to occur. Translating these considerations to our model, questions that may be helpful for discussion with psychology trainees may include: what are your experiences in supporting your family and community members?, how did you decide boundaries with folks in your close circle?, what are your experiences in crisis outside of your professional settings where there are personal relationships involved?, what are conversations you had with people within your family and community members who may have leaned on you for support?, do you involve the family members?, and how do you discuss limits to confidentiality? By integrating how to approach role conflict in providing much-needed services to family and community members outside the professional setting, training programs can support their psychology trainees while acting within legal and ethical boundaries.

Psychology trainees from collectivist cultural backgrounds may benefit from support of their own well-being as they navigate conflicts between training program expectations and community and familial obligations. Training programs should provide proactive mentoring related to role conflict and the strain dueling expectations may have on their personal well-being. Faculty advisors and fieldwork supervisors should acknowledge the potential for role conflict, proactively check-in with psychology trainees about these pressures, and offer support with cultural humility – acknowledging that standards in psychology can and should be changed to adjust to diverse perspectives and experiences. Psychology trainees may also benefit from developing informal consultation peer groups of students with similar experiences within their programs or across their professional organizations. Training programs can advocate for their psychology trainees to take on leadership positions to help support their professional organizations in adapting to address role conflict. In this way, psychology trainees who have been historically underrepresented in their fields can transform role conflict into a strength that helps them advocate within their professional organizations to legally and ethically expand the role of mental health professionals to better support family and community members.

## Rights, Responsibilities, and Welfare of All Affected Parties

When addressing the ethical standards, family and community members have the right to access mental health care and achieve health equity. Cultural norms may instill the expectation that family members support each other, and therefore, a family member with training as a psychologist would be a natural person to turn to for support. Thus, given ethical standards of social justice and beneficence, it seems clear that a psychology trainee should provide some degree of support, within their competence level, to their family members or close community members. At the same time, although psychology trainees are often aware of potential exposure to trauma and burnout when entering the profession, this ethical scenario presents new challenges to navigate. The potential role shift that occurs when functioning as a psychology trainee and a family member or close community member complicates the level of emotional commitment to a scenario. For example, in the case that a family member discloses their thoughts of suicide, a psychology trainee is left to navigate layers of emotionality, as a family member, and also decide what their course of action is as they proceed in this scenario. Does the psychology trainee conduct an informal risk assessment? Do they offer psychological first aid? Do they function as a friend with no more knowledge than a lay person who does not have proper training? Thus, given standards of non-maleficence and competence, training program support for the psychology trainee and how to approach a role conflict within a legal and ethical manner is critical for helping resolve role conflicts.

## Alternative Solutions and Consequences

If a psychology trainee were to turn down a request for support, they may bring harm to their family member, violate cultural norms, fail to advance social justice, and, ultimately cause further distress for themselves and their community. An alternative solution may include connecting the family member with available resources in the community. However, psychology trainees who experience role conflict may also be from under-resourced communities. Thus, it would be important for psychology trainees to assess available resources, determine their fit and adequacy for the family/community member and their needs, and provide support in the referral and engagement process. If available resources are inadequate, inaccessible, or not a fit for the family/community member, the psychology trainee remains stuck in role conflict.

## Resolution

**FIGURE 1:**
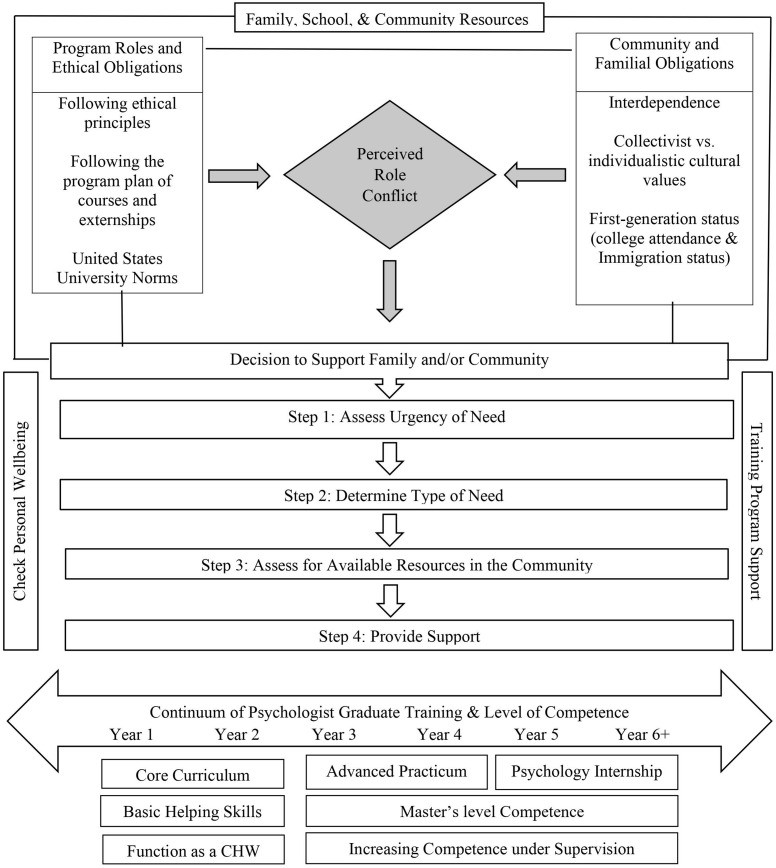
Proposed Decision-Making model for addressing role conflict for psychology trainees.

**FIGURE 2:**
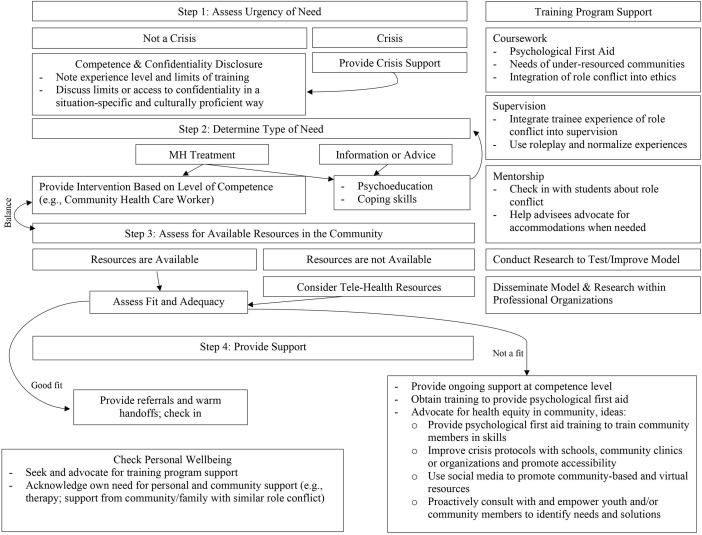
Associated flowchart for the proposed Decision-Making model for addressing role conflict for psychology trainees.

Psychology trainees gain skills that are helpful to their families and communities, especially if they are from lower-resourced backgrounds with limited accessibility. However, providing support for family or community members is a nuance in the literature that has yet to be explored. Due to the lack of literature and guidelines for supporting family or community members, it is important to gather case examples from psychology trainees who have experienced role conflict and learn how they navigated their dual roles to support the rights, responsibilities, and welfare of all affected parties. By integrating scenarios within legal and ethical guidelines of the profession, we developed a Decision-Making Model of Addressing Role Conflict for Psychology Trainees (Figure 1) and associated flow chart (Figure 2) as a first step in helping training programs and psychology trainees address role conflict. We encourage psychology trainees to consult the ethical decision-making flowchart with their supervisors if there is sufficient time. Moreover, we encourage training programs to proactively hold potential role conflict conversations with their students and offer this decision-making flowchart as a guide to support the psychology trainee’s own decision within the context of their family and community needs. It is important to note, however, that this is a conceptual model, and while grounded in theory, has not been empirically tested.

### Step One: Assess Urgency of Need

Considering the welfare of the person who may be requesting support is incredibly important when deciding how to proceed. The psychology trainee should consider whether the person is in immediate danger to themselves or others. When a family or community member is in crisis, the first step is to provide crisis support until the crisis is stabilized. In circumstances where safety may be an immediate concern, ethical standards allow psychologists to utilize their skills in case of emergency and subsequently provide resources outside of themselves. In many cases, psychologists may be tempted to involve law enforcement when safety is a major concern. However, that also presents challenges given that the involvement of law enforcement may cause more stress and escalate the mental health crisis, especially for those from low-income, rural, immigrant, and/or communities of color. The concern of involving law enforcement and potentially creating a more stressful situation for the persons involved may further encourage the psychology trainee to utilize their learned skills to support family or community members in distress.

Once the crisis is resolved, psychology trainees should also assess the repercussions of providing support versus not providing support. The psychology trainee should have a conversation with the youth (and their parent if underage) about their role as a family member or friend versus a psychology trainee; it is important to consider the long-term welfare of the relationship between the two parties. It is possible that the person requesting support depends on the psychology trainee to continue with the support. This may turn in to an unethical practice of providing continuous support without being licensed and leaving the person vulnerable to potentially incompetent services. It is the responsibility of the psychology trainee to state their level of support prior or after the interaction or as soon as they feel the informal support has reached a level of inappropriate support.

At this stage, the psychology trainee must also consider how they will protect and discuss limits of confidentiality. Due to a psychologist’s responsibility as a mandated reporter, the psychology trainee should share the limits of confidentiality prior to the interaction if possible. The informality can present a challenge in sharing limits of confidentiality due to the lack of a formal intake and consent process, where these limitations would generally be shared with the client. Thus, the psychology trainee should embed discussion of confidentiality in acceptable terms given the nature of their relationship with the family/community member seeking support. For example, it is important to discuss under what circumstances information shared will be passed along to other family or community members.

### Step Two: Determine the Type of Need

In scenarios with fewer safety concerns, for example, if a person is sharing feelings of sadness, depression, past trauma, or high anxiety, the psychology trainee can choose to provide some relief through providing psychoeducation or coping mechanisms until the person is able to see another trained professional. Additional mental health support is not optimal given dual relationships, however, support could be provided at the psychology trainee’s level of competence (e.g., CHW) depending on the availability of resources in the community.

### Step Three: Assess for Available Resources in the Community

The psychology trainee should locate the best possible mental health supports for their family member or friend. Ideally this would include a community mental health organization for the family or community member seeking support, so they are able to receive continuous support. In the event that there is no mental health support available, due to lack mental health professionals in the area or the lack of insurance to pay for services, the psychologist should share online resources that they can refer to (e.g., mental health phone applications, emergency suicide hotlines, online blogs, addiction support groups, coping strategies found online) so they are able to better support themselves. Although providing online resources does not equate to equitable mental health services and may leave the person at a disadvantage when compared to others who have readily available mental health resources, providing some guidance and resources likely leaves the client better off than without any support. Ultimately, the goal of the psychology trainee should be to support their family member or friend through the crisis and connect them with long-term support and resources.

### Step Four: Provide Support

Optimally, the psychology trainee is able to limit support to crisis intervention, psychoeducation and coping skills, and referrals and check-ins for accessing existing resources within their community. However, if ongoing support is necessary, it is important for the psychology trainee to provide support at their competence level while obtaining supervision within their training program. At early stages of their program, psychology trainees may only be able to practice at the level of a CHW. As they develop skills and obtain supervision in more advanced interventions, psychology trainees may be able to provide more significant support. However, this should be guided by cultural norms and expectations, competence and confidentiality disclosures, training program supervision, and advocacy for better access to resources in the community.

### Check Personal Wellbeing

Having training program supervision and mentorship focused on role conflict and building a community of peers who also experience role conflict may help the psychology trainee manage the stress and emotions that come with providing mental health support to family and community members. We recommend that training programs embed proactive attention to role conflict within faculty advising. Faculty advisors have key opportunities during recruitment, the initial meeting, and regular check-ins. At recruitment faculty members should identify goodness of fit with a potential advisee through questions about preferred methods of communication and feedback. At this point a faculty mentor may acknowledge challenges related to role conflict and how the psychology trainee can anticipate support and guidance for these challenges (e.g., open door policy). Once an advisee is enrolled, the initial mentoring meeting should include discussion of what the student is looking for in a mentor and how they like to receive help when challenges inevitably arise. Having a direct conversation about potential role conflict for the psychology trainee will help facilitate open dialogue about these issues when they arise. Finally, annual meetings are a good opportunity to check in about a variety of advising issues including checking in on the well-being of family and community members and how to navigate role conflict. It is critical that this support be embedded with cultural humility in that individualistic cultural values, such as prioritizing the psychology trainee’s well-being above that of their family, should not be prioritized over understanding the cultural values and expectations of the psychology trainee and their loved ones.

## Discussion

This ethical dilemma presents unique challenges in determining the extent of a psychology trainee’s ethical responsibility to help those under psychological distress within their circle of family and/or community members. The challenge becomes further exacerbated by the lack of guidance. While reviewing the APA principles and NASP standards there is little direction on providing services to a psychology trainee’s own community or family members outside the professional setting. Due to the lack of guidance in research and the standards, it is important that training programs consider the reality of students supporting family and community members in need. Psychology trainees who enter psychology graduate programs may feel a potential role conflict when trying to manage ethical standards and familial and community obligations. A graphical illustration of the potential role conflict is given in Figure 1.

To address this ethical dilemma and identify a standard of practice, we developed a Decision-Making Model of Addressing Role Conflict for Psychologists in Training as depicted in Figures 1, 2. This Model proposes a novel decision-making process to address the potential role conflict that diverse psychology trainees may experience when balancing the conflict between training standards and family and community member needs for mental health support. The proposed Model synthesizes various theories and concepts that may help facilitate insight on the experiences of diverse psychology trainees including role theory, intersectionality, collectivism and individualism, interdependence, and literature on first-generation college students. Through this exercise, our Model is designed to support psychology trainees and training programs in legally and ethically addressing role conflict. We recommend that training programs incorporate this model into the curriculum and supervision to proactively teach and supervise psychology trainees in how to ethically address role conflict. Next steps are to test the implementation of this model and monitor its impact on psychology trainees and their programs.

### Future Directions

Future directions should include updated amendments for both APA and NASP standards to best support psychology trainees. Doing so can honor the potential interdependence and collectivistic values of students coming from minoritized backgrounds. Additionally, discussions around inequitable mental health care access for low-income, immigrant, and/or communities of color should be a topic often discussed in schools and university settings. When discussing the inequitable mental health care system, university supervisors should take into consideration what this may mean for psychology trainees and their own communities. Even so, safeguarding for when these scenarios arise, such as, creating an assignment within supervision courses for students to create a list of resources in the communities they are from may be helpful for the community and the psychology trainee. Additional recommendations may include creating psychological first-aid workshops in rural and/or low-resourced communities to create community rapport among each other and also create sustainability of some mental health support when there is a lack of mental health professionals in the area. Importantly, this ethical challenge presents scenarios that psychology trainees may already be experiencing but without guidance due to the ambiguity of ethical standards and strict boundaries of training programs. This paper highlights the importance of discussing boundaries and support plans prior to these potential ambiguous scenarios. Psychology trainees would benefit from discussing this ethical challenge in supervision to devise recommendations preemptively.

Addressing role conflict for psychology trainees needs research. The field would benefit from further exploring the ways and frequency in which psychologists use their skills outside their professional setting. Additional research is needed to examine role conflict among psychology trainees to understand how the growing diversity of the field and clientele can be improved by shifting training programs from Euro-American norms. Special attention should be paid to the unique ways in which gender, potential role conflict, and familial responsibilities all intersect in the context of the presented ethical challenge. Additional research is also needed to better understand if role conflict is only experienced among psychology trainees from collectivistic, first-generation, and/or minoritized communities. We anticipate that role conflict is a phenomenon that is experienced by many helping professionals given that models of care in the United States are immersed in individualistic and Euro-American norms. Although role conflict and ambiguity are likely experienced by licensed psychologists, we focus here on psychology trainees in order to change the profession at the foundation. Moreover, the role conflict outlined throughout this paper is likely experienced by other masters-level mental health trainees while in their graduate programs (e.g., marriage and family therapist, licensed clinical social workers, masters-level school psychologists), although it is out of the scope of this paper to fully contribute to the training framework of these discipline. Future research is needed to empirically test the proposed decision-making model among psychology trainees. Research would also benefit from understanding how adaptations of the proposed decision-making model would translate to other mental health training programs such as those within masters-level training. These endeavors will provide evidence for this decision-making model or adapted versions that can optimally support psychology trainees when serving their family and communities outside their professional setting.

### Limitations

It is important to acknowledge that this conceptual framework and decision-making model adaptation is not without its limitations. The first limitation is that the recommendations provided are still entrenched in a system that values and functions under Euro-American models of care and training. Additionally, role conflict and role ambiguity have not been empirically researched among psychology trainees and therefore, we could not share empirical data that directly support our Model. We acknowledge that the perspective of individualism and collectivism is a limited view on the true complexity of culture. Therefore, future research should be conducted with specific attention to the intersections of identity and potential variability within collectivistic and individualistic communities.

## Conclusion

Although this ethical challenge presents with ambiguity, informality, and uncertainty, psychologists are trained professionals who are often left to decipher concepts that are not tangible and extremely complex. Psychologists are trained to be skilled problem solvers and should implement their knowledge in these circumstances to decide what the best course of action is given the limitations, safety concerns, and their competence. Thus, we are confident that with tools such as the Decision-Making Model of Addressing Role Conflict for Psychologists in Training, psychology trainees can confidently proceed in supporting their family and community members through crises and mental health needs, especially with the support of their training programs.

## Author Contributions

NL: conceptualization, writing, and model drafts – original draft preparation. JS: supervision, writing, model drafts, and training program specific additions. Both authors contributed to the article and approved the submitted version.

## Conflict of Interest

The authors declare that the research was conducted in the absence of any commercial or financial relationships that could be construed as a potential conflict of interest.

## Publisher’s Note

All claims expressed in this article are solely those of the authors and do not necessarily represent those of their affiliated organizations, or those of the publisher, the editors and the reviewers. Any product that may be evaluated in this article, or claim that may be made by its manufacturer, is not guaranteed or endorsed by the publisher.
